# Impact of the COVID-19 Pandemic on the Surgical Management of Head and Neck Non-Melanoma Skin Cancers in a Maxillofacial Center of Cluj-Napoca

**DOI:** 10.3390/jcm13133934

**Published:** 2024-07-04

**Authors:** Rareș Călin Roman, Cosmin Ioan Faur, Edina Gordan, Mădălina Văleanu, Mădălina Anca Moldovan

**Affiliations:** 1 Department of Oral and Maxillofacial Surgery and Implantology, Iuliu Hatieganu University of Medicine and Pharmacy, 400347 Cluj-Napoca, Romania; raresroman@yahoo.com (R.C.R.);; 2 Department of Oral Radiology, Iuliu Hatieganu University of Medicine and Pharmacy, 400347 Cluj-Napoca, Romania; 3 Faculty of Dental Medicine, Iuliu Hatieganu University of Medicine and Pharmacy, 400347 Cluj-Napoca, Romania; 4 Department of Statistics, Iuliu Hatieganu University of Medicine and Pharmacy, 400347 Cluj-Napoca, Romania

**Keywords:** head and neck cancer, skin cancer, non-melanoma skin cancer, COVID-19 pandemic

## Abstract

**Background**: The COVID-19 era has been a bleak period for both cancer and non-cancer patients, with delayed non-emergency treatments, such as for non-melanoma skin cancer (NMSC). This study aimed to evaluate how the treatment of NMSC patients was influenced by the management of the COVID-19 pandemic in an Eastern European Maxillofacial Surgery center. **Materials and Methods**: A total of 176 patients with a histopathological diagnosis of head and neck NMSC who were surgically treated in Cluj-Napoca Emergency County Hospital between 2016 and 2022 were included in this study, and divided into two samples, pre-pandemic (2016–2019) and COVID-19 (2020–2022) periods. **Results**: The pandemic presented with a decrease of 46.15% in patients’ hospitalization, with wealthy and educated patients being prevalent. Even if the waiting time for surgery was increased, the stage of cancer and preference method for reconstruction did not differ. Despite the lower addressability of NMSC patients during the pandemic, there were no changes in surgical treatment. **Conclusions**: During COVID-19, the number of patients was reduced, with a longer waiting time for surgery, but without any changes in tumor stage and treatment preferences. However, the benefit of removing a cancer tumor is higher compared to the risk of developing COVID-19 infection during hospitalization

## 1. Introduction

Non-melanoma skin cancer (NMSC) is today one of the most common malignancy cases worldwide. According to recent statistics, NMSC ranks 5th place with over 1.2 million new cases (excluding basal cell carcinoma) from a total of 20.0 million cancer diagnoses globally in 2022. North America had the highest incidence of NMSC (49.5%) followed by Europe (26.7%) [1]. Since the 1960s, the incidence of NMSC has increased from 4% to 8% each year [2]. There are multiple types of non-melanoma skin cancers, with the most common being basal cell carcinoma (BCC) and squamous cell carcinoma (SCC). It is estimated that every year, approximately 5.4 million lesions are detected in the United States. BCC is a lot more common (80% of NMSC) and less aggressive than SCC (20% of NMSC). These skin cancers develop mostly in sun-exposed areas, like the head and neck region and arms, and evolve slowly [3].

Risk factors for developing BCC or SCC include individual inherent ones regarding male gender, pale complexion, and increasing age, as well as genetic factors, immunosuppression, and chronic inflammatory disorders. Environmental risk factors relate to UV radiation, specifically UVB from chronic sun exposure, ionizing radiation, and chemical carcinogens like tobacco or occupational exposure [4,5]. On the one hand, individuals who have been previously diagnosed with BCC have a higher lifetime risk of developing another such tumor. Also, this risk varies with geographical location, with Northern European countries showing increasing incidences in younger people [6,7]. On the other hand, SCC may develop from pre-cancerous lesions like actinic keratosis, squamous cell carcinoma in situ, also called Bowen’s disease, and keratoacanthoma and, rarely, develop in scars or chronic skin sores in other areas of the body [8].

The gold-standard treatment of NMSC is surgical excision. The oncological resection margins depend on the histopathological type, dimension of the tumor, and the primary or recurrent type of tumor [9,10]. To increase the accuracy of the oncological margins, Mohs micrographic surgery and frozen sections can be used. However, most primary NMSCs are completely removed in 95% of cases using the traditional excision methods. Also, adjuvant methods, such as physical or chemical destruction, immunomodulation with topical 5% imiquimod, and systemic treatment with vismodegib can be used to reduce the risk of recurrences. Radiotherapy is reserved for patients in a more evolved stage, for which surgery is not a suitable option anymore. The five-year cure rate for such a treatment is estimated to be at 90% [11,12].

Comparatively, 57,043 people died of melanoma in 2020 worldwide, whereas NMSC accounted for 63,700 deaths, with numbers still being underreported, especially for BCC [13]. Mortality for BCC is rare (0.1%) and mostly occurs in immunocompromised patients or in the case of an aggressive histopathological pattern [14]. The 5-year relative survival rate is considered 99% in primary and 95% in recurrent BCCs [12]. SCC has a lower 5-year survival rate of around 85% for primary and 58% for advanced lesions, compared to BCC, due to the risk of loco-regional and systemic dissemination [15].

The morbidity of head and neck cancers has a considerable impact on the psychological level of the patients and their families. Modern medicine with innovative reconstructive surgery and precise excision played an important role in raising patient satisfaction, but the effects on mental health are still not negligible [16].

The COVID-19 pandemic, caused by acute respiratory syndrome coronavirus 2 (SARS-CoV-2), was officially declared on 31 March 2020 and quickly spread across the whole of Europe, with the most affected countries being Italy, Spain, France, and the UK [17]. When comparing Romania to the European average, the mortality and case fatality regarding COVID-19 infections were above average [18]. In such a global context, non-urgent cancer treatments were interrupted, and low-risk lesions were kept under observation for 3 to 6 months, prioritizing more severe cases (e.g., highly symptomatic patients with high-risk, ulcerative, perineural invasion or rapid growth patterns of NMSC) [19]. Also, patients were reluctant to seek medical care in a public hospital setting, with a drop in new head and neck cancer diagnoses of 20–30% being observed. The patients tended to wait longer before attending the hospital, with an increased symptom-to-diagnosis interval, with regional metastatic disease and complex surgery being observed [20,21]. It is well known that delayed presentation for such cancers inevitably results in poorer outcomes and a necessity for more aggressive treatment. In a Washington Hospital Center, a 50% decrease in new cases of head and neck SCC was observed, with more patients being hospitalized rather than treated in ambulatory care [22]. Also, in Modena, Italy, an increase in tumor stage at presentation was observed for patients in the first half of 2020 compared to 2019 [23]. However, data about NMSC are lacking, especially in Eastern European countries, such as Romania.

The financial burden of the coronavirus pandemic, combined with that of increasing skin cancer cases, put pressure on the already challenged healthcare system. The necessity for additional protective equipment and COVID-19 testing represented further expenses. In 2020, revenues for the Romanian Insurance and social assistance sector increased by 123.44%, while expenses increased by 140.4% [24]. With Romania being in the second place for COVID-19-related deaths in the EU in 2020 (80% of total deaths) [25], totaling up to 67,000 deaths until 2022, the challenges faced were even more significant [26].

There is no study that evaluates the changes that appeared in head and neck skin cancer in Romania during the COVID-19 pandemic, regarding epidemiology, treatment, and financial burden. We aimed to evaluate the impact of the COVID-19 pandemic on head and neck NMSC management in a National Healthcare Hospital from Cluj-Napoca.

## 2. Materials and Methods

This retrospective study included patients who were admitted to the Oral and Maxil-lofacial Surgery Department of Cluj-Napoca County Clinic between January 2016 and December 2022 with a histopathological result of either primary or recurrent head or neck skin BCC or SCC. Exclusion criteria consisted of patients that underwent radiation therapy in the head and neck area, inconsistent pathology results, or Merkel cell carcinoma, melanoma, or benign tumors. All the patients were treated as inpatients and kept under observation for at least 24 h. The treatment protocol consisted of surgical excision followed by reconstruction of the defect with a skin graft, local flap, or primary closure. The American Joint Committee on Cancer was used to stage the tumors (8th ed.) [27,28].

For an unbiased evaluation of their chronic illness, patients’ overall health condition was classified utilizing the American Society of Anesthesiologists (ASA) risk stratification. A questionnaire about education and financial status was completed only by part of the patients. This study was approved by the Ethics Committee of “Iuliu Hatieganu” University of Medicine and Pharmacy (AVZ 70/04.03.2024), and it followed the updated Declaration of Helsinki.

### 2.1. Data Collection and Statistical Analysis

We utilized a past protocol for the data collection and statistical analysis [29]. Briefly, we collected the demographical, clinical, and financial information from the release record of patients identified on Cluj-Napoca’s County Hospital’s informatics system, Atlas MED. A patient’s treated malignant skin tumor is considered an episode (of cancer). The prevalent pattern found upon histological examination represented the criteria for classification. The direct treatment costs (hospitalization accommodation and professional assistance costs, material costs, medication costs, and paraclinical examination costs) were considered, without any other costs regarding follow-up or the indirect (e.g., productivity costs) or intangible costs (e.g., the monetary value of health loss and reduction in quality of life). All the costs are stated in euros utilizing the medium exchange rate (the 5-year Euro-RON medium exchange rate) based on the Romanian National Bank rank. Moreover, as per the Romanian National Bank, the costs are adjusted to align with the rate of inflation through 2024.

### 2.2. Analysis of Statistical Data

For statistical analysis and data description, SPSS 25.0 (SPSS Inc., Chicago, IL, USA) was utilized. A statistical significance threshold of α = 0.05 was established. The mean ± standard deviation is utilized to describe normally distributed continuous quantitative data, and the median (first quartile-third quartile) for non-normally distributed quantitative data. The absolute and relative frequencies (%) are employed to describe qualitative data.

Student’s *t*-test was utilized to compare the means of the normally distributed data of two independent groups. The means of two independent groups with non-normal distributions were compared using the non-parametric Mann–Whitney and Kruskal–Wallis tests. Fisher or Chi-Square tests were used to compare qualitative variables. Univariate regression analysis was used to estimate costs.

## 3. Results

### 3.1. Demographical and Epidemiological Consideration of the Population

A total of 176 patients were included, with 63 being surgically treated in the COVID-19 period. Therefore, a decrease of 46.15% in patients’ hospitalization was observed in the COVID-19 period compared with the pre-pandemic era.

The 176 patients had 211 episodes of NMSC (166 BCC, 45 SCC), 21 patients had two or more BCC tumors, 1 had two or more SCC tumors, and 5 patients had both BCC and SCC. One 74-year-old female patient had 4 NMSCs and a 58-year-old male had 6 NMSCs surgically removed.

The median age of the patients treated for NMSC during the whole analyzed period was 73 (61–78). No statistically significant difference was observed between patients in the pandemic and pre-pandemic periods regarding age (72 (61–77) vs. 73 (65–80)) or sex (Table 1).

We observed a statistically significant difference regarding living place, education, and income (Table 1). The majority of the patients treated during the COVID-19 pandemic lived in urban places (41%), attended high school or university (56%), and had medium or high incomes (66%) compared to pre-pandemic patients.

The ASA risk evaluation for the period indicated that the majority of patients treated for head and neck NMSC were classified in class I or II (73%). The ASA risk staging suffered no changes in COVID-19 pandemic patients compared to pre-pandemic patients (Table 1).

The BCC-to-SCC ratio was 3.68:1. In the pre-pandemic period, the BCC-to-SCC ratio was 4:1, whereas in the COVID-19 period, the BCC:SCC ratio was 3.1:1 (Table 1, *p* < 0.001). The nodular subtype was the predominant BCC variant (65.06%) in both the COVID-19 period and pre-pandemic period cohorts, whereas moderately differentiated SCC (G2) was the most common subtype of SCC (51%) in the pre-COVID-19 era and well-differentiated SCC (G1) was prevalent in the COVID-19 pandemic period (*p* < 0.001).

The nasal region was commonly (27%) affected by NMSC tumors, followed by cheek and frontal NMSC tumors (Table 1). The most frequent tumor site in the pandemic period was the cheek region (26%), compared to the pre-pandemic era where the nasal region (30%) was more frequently involved by NMSC lesions. Also, the nasal region was predominantly involved by BCC (30%, 50 excisions), while the cheek region was commonly affected by SCC (26%, 12 excisions).

No statistically significant difference was observed between pre- and COVID-19 pandemic periods regarding stages of NMSC tumors (Table 1). The early stages (I and II) totaled 83% in the pre-pandemic period and 89% in the COVID-19 pandemic. Out of the more advanced stages (III and IV, 31 excisions), BCC represented 51% (16 excisions) and SCC 48% (15 excisions). No metastatic disease was observed.

The patients waited longer to be admitted to the hospital for NMSC treatment during the COVID-19 pandemic (2.44 ± 3.92 weeks) compared to the pre-pandemic period (1.31 ± 2.94, *p* = 0.004). The percentage of re-excisions was higher during the COVID-19 pandemic compared to pre-pandemic (7% vs. 2%, Table 1). However, the hospitalization and type of reconstruction did not differ (Table 1). The local flaps and primary sutures were preferred in both COVID-19 and pre-pandemic periods, with the patients being hospitalized between 1 and 16 days in the hospital, with a mean of 2.3 ± 1.92 days. However, the time for surgery was increased by 39.39% during the pandemic period, from 1.02 ± 0.87 h to 1.39 ± 0.73 h.

### 3.2. Treatment Costs

**Figure 1:**
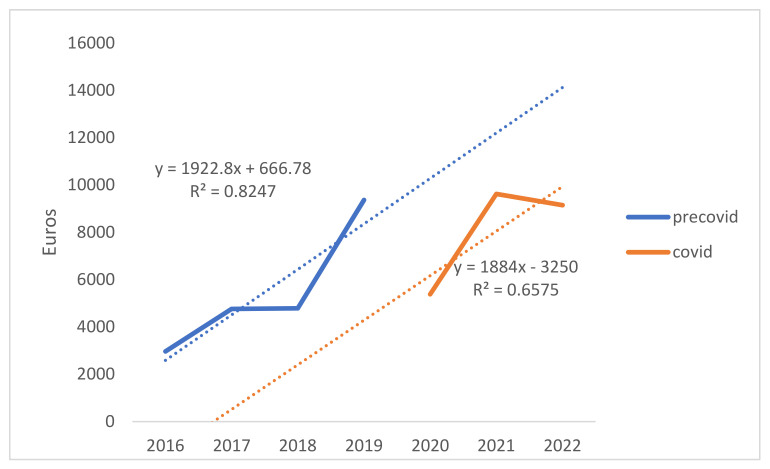
Costs analysis of COVID-19 pandemic vs. pre-pandemic periods.

The total costs for head and neck skin cancer treatment were EUR 45,120 in the pre-pandemic period and EUR 35,668 in the COVID-19 pandemic period. The mean ± standard deviation cost/episode of head and neck NMSC treatment for the whole analyzed period was EUR 371 ± 401. We observed an increased cost/episode from EUR 333 ± 311 in the pre-pandemic period to EUR 475 ± 533 in the COVID-19 pandemic period (*p* = 0.004, Table 2, Figure 1). The highest cost for skin cancer treatment was for a 76-year-old male patient (EUR 3483) who presented in 2022 for the removal of an SCC from the orbital area.

Besides accommodation costs and professional assistance, the majority of the costs were represented by examinations (13.82%) (Table 2). All the particular costs/episode (examinations, medications, materials, accommodation, and professional assistance) were higher in the COVID-19 pandemic period compared to the pre-pandemic era with an overall rise of 52.7%. During the COVID-19 pandemic, an increase of 42.04% for accommodation and professional assistance, 7.81% for medication, 163.42% for materials, and 113.49% for examinations were observed.

## 4. Discussion

Far from our knowledge, this is the first study that evaluates the impact of the COVID-19 pandemic on the management of head and neck oncological patients.

Similar to other studies, we observed fewer patients that presented to the hospital for non-urgent care during the COVID-19 pandemic [20,21]. This lack of patient referrals may be due to the COVID-19 lockdown rules, the fear of COVID-19 infection, or the healthcare system collapse. However, different types of skin cancer, such as SCC, need urgent care due to the capacity to spread through the lymphatic system and metastatic disease which may drastically reduce the survival rate [22,23].

Even if the age and sex did not differ between pre-pandemic and COVID-19 pandemic periods, access to the hospital was easier for the patients who lived in urban places. Also, the percentage of patients who attended university or high school programs or had a good economic status (medium and high) was higher in the COVID-19 period compared to the pre-pandemic period. That may be due to these patients’ education and a higher interest in their health [30,31].

The patients who were admitted to the hospital during the COVID-19 period were negative for COVID-19 in a previous test. This may explain the lack of difference between the ASA risk of patients admitted in the pre-pandemic and COVID-19 periods. Also, the time of hospitalization required for NMSC treatment was similar between the two periods, which indicated the possibility of treating head and neck skin cancer as fast-track surgery [29,32,33].

The waiting interval between the diagnosis and the surgery time was higher in the COVID-19 period compared to the pre-COVID-19 era, similar to other studies [34]. In the COVID-19 period, the hospitals had limited available beds for non-urgent, non-COVID-19-infected patients [34,35,36]. However, the stage of the disease did not differ between the two periods in our study. This was also seen in the lack of difference between pre-pandemic and COVID-19 periods for the preferred type of reconstruction, with the local flap being the first choice, followed by the primary suture after cancer excision. However, the time for surgery differed, being slightly increased during the COVID-19 period. This may be explained by the different protocols for patients’ circuits, surgeons’ preparation, and supplementary protections against COVID-19 infections [37,38,39].

The costs for NMSC of the head and neck regions were also higher in the COVID-19 period compared to the pre-COVID-19 era, similar to other studies [40]. During the COVID-19 pandemic, the number of examinations was increased as a part of the prevention protocol against COVID-19 and rapid detection of the infection [38]. Also, adjuvant materials were used to prevent the infection [41]. These protocols can be seen in the higher costs of materials and examinations during COVID-19 periods.

The present study has certain limitations that may be considered when analyzing the results. Besides the reduced number of patients, not all patients shared their income or education level. Furthermore, the particular costs for surgery cannot be retrieved from the Hospital Informatic System, due to the standard cost estimation required by the National Healthcare System Department for the payment of the treatment [29].

## 5. Conclusions

During the COVID-19 pandemic, the number of non-melanoma head and neck skin cancer patients was reduced by approximately one-third compared to the pre-pandemic period. However, the treatment standards, such as the preference for local flap reconstruction, hospitalization time, and the stage of the disease, remained unchanged. Nevertheless, the waiting time for surgery and the treatment costs for hospitalization were increased due to the increased costs of the materials and examinations that were required for COVID-19 prevention.

## Figures and Tables

**Table 1:** Epidemiology and treatment features of non-melanoma head and neck skin cancer in Cluj-Napoca County Hospital (2016–2022).

	2016–2022	Pre-Pandemic (2016–2019)	Pandemic (2020–2022)	Statistical Analysis Pre- vs. during Pandemic
Sex				
Male	101 (57.38%)	68 (58.11%)	36 (57.14%)	0.787
Female	75 (42.61%)	49 (41.88%)	27 (42.85%)	
Living place				
Urban	111 (63.06%)	69 (58.97%)	45 (41.02%)	0.028
Rural	65 (36.93%)	48 (41.02%)	18 (28.57%)	
Education				
Primary school	10 (13.33%)	5 (10%)	5 (20%)	0.003
Middle school	26 (34.66%)	20 (40%)	6 (24%)	
High school	19 (25.33%)	14 (28%)	5 (20%)	
University	20 (26.66%)	11 (22%)	9 (36%)	
Income				
Low	49 (52.68%)	38 (63.33%)	11 (33.33%)	0.037
Medium	42 (45.16%)	21 (35%)	21 (63.63%)	
High	2 (2.15%)	1 (1.66%)	1 (3.03%)	
ASA risk				
I	71 (39.01%)	43 (35.83%)	29 (45.31%)	0.099
II	60 (32.96%)	41 (34.16%)	19 (29.68%)	
III	50 (27.47%)	36 (30%)	15 (23.43%)	
IV	1 (0.54%)	0 (0%)	1 (1.56%)	
Localization				
Nasal	58 (27.48%)	42 (30.88%)	16 (21.33%)	-
Cheek	47 (22.2%)	27 (19.85%)	20 (26.67%)	
Temporal	18 (8.53%)	14 (10.29%)	4 (5.33%)	
Frontal	28 (13.27%)	16 (11.76%)	12 (16.00%)	
Auricular	17 (8.05%)	9 (6.62%)	8 (10.67%)	
Cervical	4 (1.89%)	2 (1.47%)	2 (2.67%)	
Labial	5 (2.36%)	4 (2.94%)	1 (1.33%)	
Mental	5 (2.36%)	3 (2.21%)	2 (2.67%)	
Orbital	22 (10.42%)	12 (8.82%)	10 (13.33%)	
Infraorbital	7 (3.31%)	7 (5.15%)	0 (0%)	
Type of tumor and histopathological grade				
BCC	166 (78.64%)	109 (80.14%)	57 (76%)	-
Nodular	108 (65.06%)	70 (64.22%)	38 (66.66%)	
Infiltrative	34 (20.48%)	25 (22.93%)	9 (15.78%)	
Other types	24 (14.45%)	14 (12.84%)	10 (17.54%)	
SCC	45 (21.32%)	27 (19.85%)	18 (24%)	
G1	19 (42.22%)	8 (29.62%)	11 (61.11%)	
G2	20 (44.44%)	14 (51.85%)	6 (33.33%)	
G3	4 (8.88%)	3 (11.11%)	1 (5.55%)	
G4	2 (4.44%)	2 (7.40%)	0 (0%)	
Patients with both BCC and SCC	5 (2.84%)	5 (4.27%)	1 (1.58%)	
Stage				
I	136 (64.45%)	85 (62.5%)	51 (68%)	0.677
II	44 (20.86%)	28 (20.58%)	16 (21.33%)	
III	27 (12.79%)	20 (14.70%)	7 (9.33%)	
IV	4 (1.89%)	3 (2.20%)	1 (1.33%)	
Waiting time for surgery (weeks)				
	1.71 ± 3.36	1.31 ± 2.94	2.44 ± 3.92	0.004
Type of surgery				
Excision	202 (95.73%)	132 (97.05%)	70 (93.33%)	0.354
Re-excision	9 (4.26%)	4 (2.94%)	5 (6.66%)	
Type of reconstruction				
Primary suture	74 (35.07%)	47 (34.55%)	27 (36%)	0.354
Local flap	129 (61.13%)	87 (63.97%)	42 (56%)	
Skin graft	8 (3.79%)	2 (1.47%)	6 (8%)	
Time for surgery (hours)				
	1.15 ± 0.84	1.02 ± 0.87	1.39 ± 0.73	<0.001
Hospitalization (days)				
	2.3 ± 1.92	2.3 ± 1.77	2.31 ± 2.19	0.749

**Table 2:** Treatment costs (mean ± standard deviation) of non-melanoma head and neck skin cancer episode in Cluj-Napoca County Hospital (2016–2022).

	2016–2022	Pre-Pandemic (2016–2019)	Pandemic (2020–2022)	Statistical Analysis Pre- vs. during Pandemic
Total cost of treatment	378.85 ± 409.92	333.96 ± 311.5	475.57 ± 533.3	<0.001
Examination costs	45.48 ± 43.54	35.00 ± 34.93	65.75 ± 49.95	<0.001
Costs of medication	16.97 ± 44.87	17.23 ± 53.46	17.43 ± 29.77	0.002
Costs of materials	22.23 ± 29.90	14.67 ± 17.05	36.29 ± 40.44	<0.001
Accomodation and professional assistance	294.17 ± 328.74	267.06 ± 234.76	356.10 ± 445.47	0.004

## Data Availability

The data presented in this study are available on request from the corresponding author due to data confidentiality of patients.

## References

[1] Bray F., Laversanne M., Sung H., Ferlay J., Siegel R.L., Soerjomataram I., Jemal A. (2024). Global Cancer Statistics 2022: GLOBOCAN Estimates of Incidence and Mortality Worldwide for 36 Cancers in 185 Countries. CA Cancer J. Clin..

[2] Mudigonda T., Pearce D.J., Yentzer B.A., Williford P., Feldman S.R. (2010). The Economic Impact of Non-Melanoma Skin Cancer: A Review. J. Natl. Compr. Cancer Netw..

[3] The American Cancer Society Key Statistics for Basal and Squamous Cell Skin Cancers. https://www.cancer.org/cancer/types/basal-and-squamous-cell-skin-cancer/about/key-statistics.html#:~:text=According%20to%20one%20estimate%2C%20about,cell%20cancers%20occur%20less%20often.

[4] Roland N., Memon A. (2023). Non-Melanoma Skin Cancer of the Head and Neck. Br. J. Hosp. Med..

[5] Peris K., Fargnoli M.C., Kaufmann R., Arenberger P., Bastholt L., Seguin N.B., Bataille V., Brochez L., del Marmol V., Dummer R. (2023). European Consensus-Based Interdisciplinary Guideline for Diagnosis and Treatment of Basal Cell Carcinoma—Update 2023. Eur. J. Cancer.

[6] Verkouteren J.A.C., Ramdas K.H.R., Wakkee M., Nijsten T. (2017). Epidemiology of Basal Cell Carcinoma: Scholarly Review. Br. J. Dermatol..

[7] Flohil S.C., van der Leest R.J.T., Arends L.R., de Vries E., Nijsten T. (2013). Risk of Subsequent Cutaneous Malignancy in Patients with Prior Keratinocyte Carcinoma: A Systematic Review and Meta-Analysis. Eur. J. Cancer.

[8] The American Cancer Society What Are Basal and Squamous Cell Skin Cancers?. https://www.cancer.org/cancer/types/basal-and-squamous-cell-skin-cancer/about/what-is-basal-and-squamous-cell.html.

[9] Quazi S.J., Aslam N., Saleem H., Rahman J., Khan S. (2020). Surgical Margin of Excision in Basal Cell Carcinoma: A Systematic Review of Literature. Cureus.

[10] Bichakjian C.K., Olencki T., Aasi S.Z., Alam M., Andersen J.S., Berg D., Bowen G.M., Cheney R.T., Daniels G.A., Glass L.F. (2016). Basal Cell Skin Cancer, Version 1.2016, NCCN Clinical Practice Guidelines in Oncology. J. Natl. Compr. Cancer Netw..

[11] Barker C.A., Arron S., Ho A., Algazi A., Dunn L., Humphries A., Hultman C., Lian M., Knott P.D., Yom S.S. (2024). A Phase II Single Arm Trial of Induction and Concurrent Vismodegib with Curative Intent Radiation Therapy for Locally Advanced, Unresectable Basal Cell Carcinoma of the Head and Neck. Int. J. Radiat. Oncol. Biol. Phys..

[12] Wong C.S.M., Strange R.C., Lear J.T. (2003). Basal Cell Carcinoma. BMJ.

[13] Samir S., Puneet K., Deepthi B., Laurence P.-D., Delphine K., Thierry P. (2023). A Comprehensive Analysis of Global Skin Cancer Incidence and Mortality with a Focus on Dermatologist Density and Population Risk Factors. https://s3.eu-central-1.amazonaws.com/m-anage.com.storage.eadv/abstracts_congress2023/37072.pdf.

[14] Flohil S.C., Seubring I., van Rossum M.M., Coebergh J.-W.W., de Vries E., Nijsten T. (2013). Trends in Basal Cell Carcinoma Incidence Rates: A 37-Year Dutch Observational Study. J. Investig. Dermatol..

[15] Stratigos A.J., Garbe C., Dessinioti C., Lebbe C., van Akkooi A., Bataille V., Bastholt L., Dreno B., Dummer R., Fargnoli M.C. (2023). European Consensus-Based Interdisciplinary Guideline for Invasive Cutaneous Squamous Cell Carcinoma. Part 1: Diagnostics and Prevention—Update 2023. Eur. J. Cancer.

[16] De Boer M.F., McCormick L.K., Pruyn J.F.A., Ryckman R.M., van den Borne B.W. (1999). Physical and Psychosocial Correlates of Head and Neck Cancer: A Review of the Literature. Otolaryngol. Head Neck Surg..

[17] Pillai S., Siddika N., Hoque Apu E., Kabir R. (2020). COVID-19: Situation of European Countries so Far. Arch. Med. Res..

[18] Enciu B.G., Tănase A.A., Drăgănescu A.C., Aramă V., Pițigoi D., Crăciun M.-D. (2022). The COVID-19 Pandemic in Romania: A Comparative Description with Its Border Countries. Healthcare.

[19] Rashid S., Tsao H. (2021). Effect of the COVID-19 Pandemic on Delayed Skin Cancer Services. Dermatol. Clin..

[20] Kourtidis S., Münst J., Hofmann V.M. (2022). Effects of the COVID-19 Pandemic on Head and Neck Cancer Stage and Treatment Duration. Cureus.

[21] Flores C.E.R., de Almeida D.O., Freitas L.V.D., Martins I.S., Martins N.M.B., Danesi C.C., Ferrazzo K.L. (2023). Diagnosis of head and neck squamous cell carcinoma during the COVID-19 pandemic: A retrospective case-control study. Oral. Surg. Oral. Med. Oral. Pathol. Oral. Radiol..

[22] Crossley J.R., Nelson L.L., VanDolah H., Davidson B.J., Maxwell J.H. (2022). The Impact of COVID-19 on Presentation and Diagnosis of Head and Neck Squamous Cell Carcinoma. Laryngoscope Investig. Otolaryngol..

[23] Lucidi D., Valerini S., Federici G., Miglio M., Cantaffa C., Alicandri-Ciufelli M. (2022). Head and Neck Cancer During COVID-19 Pandemic: Was There a Diagnostic Delay?. Indian J. Otolaryngol. Head Neck Surg..

[24] Antohi V.M., Ionescu R.V., Zlati M.L., Mirica C. (2022). Approaches Related to the Effects of COVID-19 Pandemics on Financing of the Healthcare System in Romania. Front. Public Health.

[25] Eurostat (2020). Main Cause of Death. https://ec.europa.eu/eurostat/web/products-eurostat-news/w/DDN-20230307-3.

[26] Worldometers Coronavirus Cases. https://www.worldometers.info/coronavirus/.

[27] Zanoni D.K., Patel S.G., Shah J.P. (2019). Changes in the 8th Edition of the American Joint Committee on Cancer (AJCC) Staging of Head and Neck Cancer: Rationale and Implications. Curr. Oncol. Rep..

[28] Amin M.B., Edge S.B., Greene F.L., Byrd D.R., Brookland R.K., Washington M.K., Gershenwald J.E., Compton C.C., Hess K.R., Sullivan D.C., AJCC (2016). AJCC Cancer Staging Manual.

[29] Faur C.I., Moldovan M.A., Văleanu M., Rotar H., Filip L., Roman R.C. (2023). The Prevalence and Treatment Costs of Non-Melanoma Skin Cancer in Cluj-Napoca Maxillofacial Center. Medicina.

[30] Raghupathi V., Raghupathi W. (2020). The Influence of Education on Health: An Empirical Assessment of OECD Countries for the Period 1995–2015. Arch. Public Health.

[31] Zhang S., Xiang W. (2019). Income Gradient in Health-Related Quality of Life—The Role of Social Networking Time. Int. J. Equity Health.

[32] Benedetti S., Frosolini A., Catarzi L., Marsiglio A., Gennaro P., Gabriele G. (2024). Impact of the COVID-19 Pandemic on the Diagnosis and Management of Non-Melanoma Skin Cancer in the Head and Neck Region: A Retrospective Cohort Study. Healthcare.

[33] Lembo F., Cecchino L., Parisi D., Portincasa A. (2022). Nonmelanoma Skin Cancer in COVID-19 Era: The Foggia Experience. J. Cutan. Aesthet. Surg..

[34] Silvia C., Denis C., Mario C., Luigi V., Federico T., Marcello C. (2022). Impact of COVID-19 Pandemic on Non-Melanoma Skin Cancer’s Tumor Burden and Care: A Multi-Center Study Based in Northern Italy. J. Plast. Reconstr. Aesthet. Surg..

[35] Bartlett D.L., Howe J.R., Chang G., Crago A., Hogg M., Karakousis G., Levine E., Maker A., Mamounas E., McGuire K. (2020). Management of Cancer Surgery Cases During the COVID-19 Pandemic: Considerations. Ann. Surg. Oncol..

[36] Søreide K., Hallet J., Matthews J.B., Schnitzbauer A.A., Line P.D., Lai P.B.S., Otero J., Callegaro D., Warner S.G., Baxter N.N. (2020). Immediate and Long-Term Impact of the COVID-19 Pandemic on Delivery of Surgical Services. Br. J. Surg..

[37] Souza A.F., de Arruda J.A.A., Costa F.P.D., Bemquerer L.M., Castro W.H., Campos F.E.B., Kakehasi F.M., Travassos D.V., Silva T.A. (2021). Safety Protocols for Dental Care during the COVID-19 Pandemic: The Experience of a Brazilian Hospital Service. Braz. Oral. Res..

[38] Givi B., Schiff B.A., Chinn S.B., Clayburgh D., Iyer N.G., Jalisi S., Moore M.G., Nathan C.-A., Orloff L.A., O’Neill J.P. (2020). Safety Recommendations for Evaluation and Surgery of the Head and Neck During the COVID-19 Pandemic. JAMA Otolaryngol. Head Neck Surg..

[39] Moore D., Gamage B., Bryce E., Copes R., Yassi A. (2005). Protecting Health Care Workers from SARS and Other Respiratory Pathogens: Organizational and Individual Factors That Affect Adherence to Infection Control Guidelines. Am. J. Infect. Control.

[40] Chambers L.C. (2023). Long-Term Health Care Costs Following COVID-19: Implications for Pandemic Preparedness. Am. J. Manag. Care.

[41] Dancer S.J. (2021). Reducing the Risk of COVID-19 Transmission in Hospitals: Focus on Additional Infection Control Strategies. Surgery.

